# Prevalence and associations of microalbuminuria in proteinuria-negative patients with type 2 diabetes in two regional hospitals in Cameroon: a cross-sectional study

**DOI:** 10.1186/s13104-017-2804-5

**Published:** 2017-09-12

**Authors:** Nelsy T. Efundem, Jules Clement N. Assob, Vitalis F. Feteh, Simeon-Pierre Choukem

**Affiliations:** 1 Department of Medical Laboratory Science, Faculty of Health Sciences, University of Buea, Buea, Cameroon; 2 Health and Human Development (2HD) Research Network, Douala, Cameroon; 3 Department of Internal Medicine and Pediatrics, Faculty of Health Sciences, University of Buea, Buea, Cameroon; 4 Diabetes and Endocrine Unit, Department of Internal Medicine, Douala General Hospital, P.O. Box 4856, Douala, Cameroon

**Keywords:** Microalbuminuria, Diabetes, Diabetic nephropathy, Glomerular filtration rate, Cameroon

## Abstract

**Background:**

Microalbuminuria (MA) is the earliest clinical evidence of diabetic nephropathy, but most patients in sub-Saharan Africa (SSA) only have access to much cheaper dipstick proteinuria as a means to screen for diabetic nephropathy. The aim of this study was to determine the prevalence and associations of MA among proteinuria-negative type 2 diabetic patients in a SSA setting.

**Methods:**

In this cross-sectional study, patients with type 2 diabetes screened negative for dipstick proteinuria in a primary healthcare hospital were assessed. Detection of microalbuminuria was carried out in two steps: qualitative detection using special microalbumin urine strip, and quantitative laboratory measurement and calculation of urinary albumin-to-creatinine ratio (UACR). Microalbuminuria was defined as UACR of 30–300 mg/g.

**Results:**

A total of 162 type 2 diabetic patients were included. Using quantitative assessment, the prevalence of microalbuminuria was 14.2% (95% CI 8.8–19.6) whereas 26.5% (95% CI 19.8–34.0) had microalbuminuria with urine strip. The mean systolic blood pressure (p = 0.032), diastolic blood pressure (p = 0.032) and serum creatinine concentration (p < 0.001) were higher in people with microalbuminuria as compared to those with normoalbuminuria, whereas the mean body mass index (p = 0.046) and mean eGFR (p < 0.001) were lower in the albuminuria group. In multiple linear regression, eGFR (p = 0.001) and serum creatinine concentration (p = 0.003) were independently associated with increased UACR.

**Conclusions:**

One in every seven proteinuria-negative type 2 diabetic patients has microalbuminuria in primary care setting in Cameroon; microalbuminuria is associated with higher systolic and diastolic blood pressure, and declining kidney function. Our results emphasize the urgent need to increase the accessibility to microalbuminuria testing to ensure that all diabetic patients with negative dipstick proteinuria can benefit.

## Background

Diabetic nephropathy (DN) is a kidney disease characterized by persistent albuminuria, progressive decline in glomerular filtration rate (GFR) and raised arterial blood pressure [1]. Diabetic nephropathy is a worldwide public health problem, and it is the leading cause of end-stage renal disease (ESRD) in most parts of the developed world [2]. It is associated with increased cardiovascular mortality [3]. Approximately one-third to half of patients with diabetes develops renal manifestations [4]. In Cameroon, diabetic nephropathy is the second leading cause of end stage renal disease and dialysis [5].

Microalbuminuria, an early marker of diabetic nephropathy can progress to macroalbuminuria and ESRD [6–8] but early screening, medical treatment and appropriate lifestyle modifications have been shown to halt or reverse the progression from micro- to macroalbuminuria [8]. Hence, it is recommended that microalbuminuria be screened in all diabetic patients annually [9]. Studies in Africa have reported prevalence of microalbuminuria in diabetic patients ranging from 10 to 44% [10, 11] and up to 61% in the United Arab Emirates [12]. In Cameroon, Sobngwi et al. in 1999 reported microalbuminuria prevalence of 53.1% amongst diabetic patients in a tertiary care hospital, which was strongly associated with diabetic retinopathy [13]. Despite these data, access to routine microalbuminuria testing in sub-Saharan Africa (SSA) is hindered by costs, thus, most patients with diabetes perform dipstick proteinuria only.

The aim of this study was to determine the prevalence of microalbuminuria and to identify associated factors, in patients with type 2 diabetes who screened negative for dipstick proteinuria, in the two regional hospitals of the South West Region of Cameroon.

## Methods

### Study design, setting and population

This was a hospital-based cross-sectional study conducted at the diabetic clinics of Buea and Limbe regional hospitals. These are the two regional hospitals of the South West Region of Cameroon. Consenting participants coming for routine diabetes follow-up were consecutively recruited. Patients who presented with a febrile illness, overt proteinuria, urinary tract infection, pregnancy, diagnosed or suspected renal, hepatic or other systemic disease were excluded from the study. Those who had done intensive physical activity within the preceding 72 h were ask to come back after 72 h without intensive exercise, for inclusion.

### Data collection

#### Clinical assessment

A data collection form was used for each patient. The principal investigator collected data on medical and family history; the weight, height and blood pressure were measured using standard methods. The body mass index (BMI) was calculated as weight (kg)/[height (m)]^2^ and classified based on WHO classification [14].

#### Laboratory procedures

##### Urine collection and transportation

A second morning mid-stream urine sample (10 mL) was collected in a container with no preservatives. Collected urine was put immediately into a cooler containing ice pack, and transported to the laboratory for analysis. Samples not analysed on the day of collection were stored at 2–4 °C.

##### Urine screening

During analysis, urine samples were first screened for any urinary indicator of urinary tract infection (leukocytes, nitrites), overt proteinuria and glycosuria using ACON^®^ Urinalysis Reagent Strips with 11 parameters. Participants whose samples tested positive for leukocytes, nitrites or glycosuria were treated and urine recollected after 3 weeks, while those with overt proteinuria were excluded. The same urine sample used to assess for proteinuria was used to assess for microalbuminuria. All proteinuric patients tested positive for albuminuria, and they were excluded from the study.

##### Assessment for microalbuminuria

Two screening methods (qualitative and quantitative analysis) were used to screen for microalbuminuria using the same urine sample. Qualitatively, special microalbumin semi-quantitative urine-testing strips (CYBOW™2MAC) were used to test for microalbuminuria according to manufacturer’s instructions [15].

Quantitatively, microalbuminuria was assessed by determining urine albumin-to-creatinine ratio (UACR) from values obtained from measurements of urine creatinine and urine albumin. Urine creatinine was analysed using the modified kinetic Jaffé method while urine albumin was detected by using acid precipitation reaction between urine albumin and 12% trichloroacetic acid. The turbidity of the precipitate formed was measured spectrophotometrically. UACR was calculated and categorised as microalbuminuria if it was between 30–300 mg/g and normoalbuminuria if it was <30 mg/g based on the National Kidney Foundation Kidney Disease Outcomes Quality Initiative (NKF/KDOQI) guidelines [16]. A second urine sample was collected from those who presented with microalbuminuria (UACR between 30 and 300 mg/g).

#### Other biochemical assessments and calculations

Serum total cholesterol, high density lipoprotein-cholesterol (HDLc), triglycerides, serum creatinine, and plasma glucose were measured by spectrophotometry and expressed in mg/dL. Low density lipoprotein-LDL (LDLc) was calculated using the Friedewald equation. Renal function was assessed using the estimated glomerular filtration rate (eGFR) calculated from the modification of diet in renal disease (MDRD) formula [17].

### Sample size estimation

The minimum sample size (n) was calculated using the following formula: ${\text{n}} = {\text{p q }}\left( {{\text{Z}}_{\alpha / 2} /{\text{E}}} \right)^{ 2}$ where p was the expected prevalence, assumed to be 10.7% [11], E represents the maximum error of estimate set at 5% (0.05), for a confidence interval (CI) of 95% and Z_α/2_ is a constant dependent on the CI, 1.96. And q = 1−p (0.873). Computing the foregoing yields a minimum sample size of 147 participants.

### Data management and analysis

Data were analyzed using SPSS (Statistical Package for Social Sciences) Version 17.0 and STATA version 10.1. Results are presented as counts, percentages, means and standard deviations where applicable. Factors associated with microalbuminuria were investigated using linear regressions. A p value <0.05 was considered statistically significant.

## Results

### General characteristics of participants

Of the 162 consenting participants included, 67.3% (109) were females. Participants’ age ranged from 24 to 70 years, with mean age of 55.3 ± 10.2 years (Table 1). More than a third of the participants (36%) were in the age group of 50–59 years.

**Table 1:** Comparison of characteristics of patients with microalbuminuria and those without

Parameter	Normoalbuminuria n (%)	Microalbuminuria n (%)	p value
Sex			0.464
Male, n (%)	47 (88.7)	6 (11.3)	
Female, n (%)	92 (84.4)	17 (15.6)	
Age (years)	55.1 ± 10.5	56.30 ± 8.3	0.302
Known duration of diabetes (years)	6.1 ± 6.4	6.3 ± 5.2	0.458
BMI (kg/m^2^)	29.8 ± 0.5	27.6 ± 0.8	0.046*
SBP (mmHg)	142.2 ± 21.1	151.8 ± 28.4	0.032*
DBP (mmHg)	89.0 ± 19.6	97.2 ± 18.5	0.032*
Fasting capillary glucose (mg/dL)	151.1 ± 4.3	156.0 ± 12.7	0.676
HDLc (mg/dL)	76.5 ± 2.7	76.9 ± 5.4	0.478
LDLc (mg/dL)	75.2 ± 40.2	69.63 ± 39.6	0.538
Triglycerides (mg/dL)	123.9 ± 5.9	133.8 ± 10.8	0.257
Total cholesterol (mg/dL)	173.9 ± 4.2	167.54 ± 9.7	0.569
Serum creatinine (mg/dL)	0.9 ± 0.03	1.6 ± 0.1	<0.001*
eGFR (mL/min/1.7 m^2^)	101.3 ± 3.9	46.5 ± 2.3	<0.001*

*BMI* body mass index, *DBP* diastolic blood pressure, *eGFR* estimated glomerular filtration rate, *HDLc* high density lipoprotein cholesterol, *LDLc* low density lipoprotein cholesterol, *SPB* systolic blood pressure

* Statistically significant (p value <0.05)

### Prevalence of microalbuminuria

Using qualitative special microalbuminuria urine dipsticks, 43 participants [26.5% (95% CI 19.8–34.0)] were positive for microalbuminuria, while quantitative analyses showed that 23 participants [14.2% (95% CI 8.8–19.6%)] had microalbuminuria (UACR 30–300 mg/g). In 28 participants [17.3% (95% CI 7.9–26.8%)], the eGFR was <60 mL/min/1.73 m^2^.

### Associations with microalbuminuria

The mean systolic blood pressure (p = 0.032), diastolic blood pressure (p = 0.032) and serum creatinine concentration (p < 0.001) were higher in people with microalbuminuria as compared to those with normoalbuminuria (Table 1). The mean BMI (p = 0.046) and mean eGFR (p < 0.001) in the microalbuminuric group were lower when compared with the normoalbuminuric group (Table 1).

In a bivariate linear regression, microalbuminuria was associated with higher systolic blood pressure (p = 0.008), higher diastolic blood pressure (p = 0.015), higher serum creatinine (p < 0.001) and lower eGFR (p < 0.001) as shown in Table 2.

**Table 2:** Linear regression analysis for associations with microalbuminuria

Variables	Slope coefficient	95% CI	p
SBP	0.39	0.10 to 0.68	0.008*
DBP	0.43	0.09 to 0.77	0.015*
Serum creatinine	75.71	63.17 to 88.25	<0.001*
eGFR	−0.33	−0.46 to −0.21	<0.001*

* Statistically significant (p value <0.05)

*DBP* diastolic blood pressure, *eGFR* estimated glomerular filtration rate, *SBP* systolic blood pressure

In multivariable linear regression, only eGFR (p = 0.001) and serum creatinine concentration (p = 0.003) were independently associated with increased UACR (Table 3).

**Tabel 3:** Multiple regression analysis for associations with microalbuminuria

Variables	Slope coefficient	95% CI	p
SBP	0.11	−0.15 to 0.36	0.41
DBP	0.06	−0.24 to −0.36	0.69
eGFR	−0.3	−0.45 to −0.15	0.001*
Serum creatinine	103.24	84.00 to 122.09	0.003*

* Statistically significant (p value <0.05)

*DBP* diastolic blood pressure, *eGFR* estimated glomerular filtration rate, *SBP* systolic blood pressure

## Discussion

In this study, we found that 14.2% of non-proteinuric type 2 diabetic patients had microalbuminuria, which was associated with elevated blood pressure and declining kidney function. Reported prevalence of microalbuminuria in patients with diabetes varies widely, ranging from 10 to 61% [10–13, 18, 19], and this is probably attributable to differences in screening methods, diagnostic criteria, known duration of diabetes, the degree of control of other cardiovascular risk factors and ethnicity of study populations. The Buea and Limbe regional hospitals of the South west region of Cameroon were selected for this study due to proximity and also because such a study had never been done in these populations. However, an earlier study by Sobngwi et al. [13] on microalbuminuria in Cameroonians with diabetes included only patients from a tertiary hospital in Yaoundé in the Centre region of Cameroon and the reported prevalence was 53%, this difference in prevalence is partly explained by the high burden of microvascular complications often seen in tertiary care hospital attendees, relative to primary care settings as was the case in our study.

It has been shown that, with the presence of microalbuminuria, glomerular filtration declines by an average of 10–12 mL/min/year, and is accelerated by hypertension, though it is potentially reversible [6, 20]. Mogensen et al. reported a significant increase in cardiovascular and total mortality in subjects with type 2 diabetes who had microalbuminuria [21]. Hypertension leads to increased glomerular filtration pressure, thereby promoting abnormal glomerular permeability that enables albumin ultrafiltration [21]. Similar to most studies [10, 11, 22], this study confirms that microalbuminuria is associated with elevated systolic and diastolic blood pressures, thus emphasizing the importance of optimal blood pressure control [23] and underscoring the potential importance of routine MA screening especially in diabetic patients with coincident hypertension.

Smulders et al. reported that diabetic dyslipidemia (high serum triglyceride and low HDL cholesterol levels) is a predictor of rapid progression of microalbuminuria in patients with well-controlled blood pressure. However, like in other studies [11, 22, 24], we found no significant correlation between microalbuminuria and serum triglycerides and cholesterol levels.

Poor glycemic control favors the progression of diabetes complications [25]. Although not statistically significant in this study, patients with microalbuminuria had higher fasting blood glucose levels, compared to their normoalbuminuric counterparts. Prospective diabetes trials have proved the importance of optimal glycemic control in preventing the occurrence and progression of diabetic complications including microalbuminuria [26].

It is evident that microalbuminuria is a significant problem amongst Cameroonian type 2 diabetic patients in a primary care setting and as such routine screening for microalbuminuria which heralds severe renal dysfunction should be instituted universally in Cameroon. This is important because subtle derangements in kidney function (like microalbuminuria) are potentially preventable and/or reversible by judicious use of angiotesin-converting-enzyme inhibitors for optimal blood pressure control in type to diabetic patients [27].

We acknowledge the following potential limitations: the role angiotensin converting enzyme inhibitor or angiotensin receptor blockers on prevalence of microalbuminuria was not assessed. Also, we could not use HbA1c to evaluated glycemic control because it is not available in these regional hospitals. However, this study to the best of our knowledge is amongst the rare studies in Cameroon that has assessed epidemiology of microalbuminuria in diabetic patients with negative dipstick proteinuria in a primary healthcare setting.

## Conclusions

One in every seven proteinuria-negative type 2 diabetic patients attending the out-patient diabetic clinics in a primary care setting in Cameroon has microalbuminuria. Microalbuminuria is associated with elevated blood pressure and low eGFR. This study emphasises the urgent need to increase the access to microalbuminuria testing so as to allow all diabetic patient with negative dipstick proteinuria to benefit. It also raises the importance of optimal blood pressure control in diabetic patients so as to prevent associated renal and cardiovascular complications.

## Abbreviations

BMI: body mass index
CI: confidence interval
DN: diabetic nephropathy
eGFR: estimated glomerular filtration rate
ESRD: end stage renal disease
HDLc: high density lipoprotein cholesterol
LDLc: low density lipoprotein cholesterol
MDRD: modification of diet in renal disease
SSA: Sub-Saharan Africa
UACR: urinary albumin-to-creatinine ratio

## References

[1] Obineche EN, Adem A (2005). Update in diabetic nephropathy. Int J Diabetes Metab.

[2] Coresh J, Selvin E, Stevens LA (2007). Prevalence of chronic kidney disease in the united states. JAMA.

[3] Valmadrid CT, Klein R, Moss SE, Klein BE (2000). The risk of cardiovascular disease mortality associated with microalbuminuria and gross proteinuria in persons with older-onset diabetes mellitus. Arch Intern Med.

[4] Pecoits-Filho R, Abensur H, Betônico CCR, Machado AD, Parente EB, Queiroz M (2016). Interactions between kidney disease and diabetes: dangerous liaisons. Diabetol Metab Syndr.

[5] Halle MP, Takongue C, Kengne AP, Kaze FF, Ngu KB. Epidemiological profile of patients with end stage renal disease in a referral hospital in Cameroon. BMC Nephrol. 2015;16. http://www.ncbi.nlm.nih.gov/pmc/articles/PMC4413994/. Accessed 9 Mar 2016. 10.1186/s12882-015-0044-2 PMC4413994 25896605

[6] Mogensen CE (1984). Microalbuminuria predicts clinical proteinuria and early mortality in maturity-onset diabetes. N Engl J Med.

[7] Giorgino F, Laviola L, Perin PC, Solnica B, Fuller J, Chaturvedi N (2004). Factors associated with progression to macroalbuminuria in microalbuminuric type 1 diabetic patients: the EURODIAB Prospective Complications Study. Diabetologia.

[8] Bakris GL, Molitch M (2014). Microalbuminuria as a risk predictor in diabetes: the continuing saga. Diabetes Care.

[9] Sacks DB, Arnold M, Bakris GL, Bruns DE, Horvath AR, Kirkman MS (2011). Position statement executive summary: guidelines and recommendations for laboratory analysis in the diagnosis and management of diabetes mellitus. Diabetes Care.

[10] Eghan BA, Frempong MT, Adjei-Poku M (2007). Prevalence and predictors of microalbuminuria in patients with diabetes mellitus: a cross-sectional observational study in Kumasi, Ghana. Ethn Dis.

[11] Lutale JJK, Thordarson H, Abbas ZG, Vetvik K (2007). Microalbuminuria among type 1 and type 2 diabetic patients of African origin in Dar Es Salaam, Tanzania. BMC Nephrol.

[12] Al-Maskari F, El-Sadig M, Obineche E (2008). Prevalence and determinants of microalbuminuria among diabetic patients in the United Arab Emirates. BMC Nephrol.

[13] Sobngwi E, Mbanya JC, Moukouri EN, Ngu KB (1999). Microalbuminuria and retinopathy in a diabetic population of Cameroon. Diabetes Res Clin Pract.

[14] WHO. Physical status: the use and interpretation of anthropometry. WHO. http://www.who.int/childgrowth/publications/physical_status/en/. Accessed 5 May 2016.

[15] Sacks DB, Arnold M, Bakris GL, Bruns DE, Horvath AR, Kirkman MS (2011). Guidelines and recommendations for laboratory analysis in the diagnosis and management of diabetes mellitus. Diabetes Care.

[16] National Kidney Foundation (2002). K/DOQI clinical practice guidelines for chronic kidney disease: evaluation, classification, and stratification. Am J Kidney Dis Off J Natl Kidney Foundation.

[17] Levey AS, Bosch JP, Lewis JB, Greene T, Rogers N, Roth D (1999). A more accurate method to estimate glomerular filtration rate from serum creatinine: a new prediction equation. Ann Intern Med.

[18] Wanjohi FW, Otieno FCF, Ogola EN, Amayo EO (2002). Nephropathy in patients with recently diagnosed type 2 diabetes mellitus in black Africans. East Afr Med J.

[19] Weir MR (2004). Albuminuria predicting outcome in diabetes: incidence of microalbuminuria in Asia-Pacific Rim. Kidney Int.

[20] Ritz E, Orth SR (1999). Nephropathy in patients with type 2 diabetes mellitus. N Engl J Med.

[21] Mogensen CE (2003). Microalbuminuria and hypertension with focus on type 1 and type 2 diabetes. J Intern Med.

[22] Afkhami-Ardekani M, Modarresi M, Amirchaghmaghi E (2008). Prevalence of microalbuminuria and its risk factors in type 2 diabetic patients. Indian J Nephrol.

[23] Choukem S-P, Dzudie A, Dehayem M, Halle M-P, Doualla M-S, Luma H (2012). Comparison of different blood pressure indices for the prediction of prevalent diabetic nephropathy in a sub-Saharan African population with type 2 diabetes. Pan Afr Med J.

[24] Mather HM, Chaturvedi N, Kehely AM (1998). Comparison of prevalence and risk factors for microalbuminuria in South Asians and Europeans with type 2 diabetes mellitus. Diabet Med.

[25] Bilous R (2008). Microvascular disease: what does the UKPDS tell us about diabetic nephropathy?. Diabet Med.

[26] de Boer IH (2014). Kidney disease and related findings in the diabetes control and complications trial/epidemiology of diabetes interventions and complications study. Diabetes Care.

[27] Ruggenenti P, Fassi A, Ilieva AP, Bruno S, Iliev IP, Brusegan V (2004). Preventing microalbuminuria in type 2 diabetes. N Engl J Med.

